# Haskap Berry Leaves (*Lonicera caerulea* L.)—The Favorable Potential of Medical Use

**DOI:** 10.3390/nu14193898

**Published:** 2022-09-21

**Authors:** Szymon Sip, Anna Sip, Piotr Szulc, Judyta Cielecka-Piontek

**Affiliations:** 1 Department of Pharmacognosy, Faculty of Pharmacy, Poznań University of Medical Sciences, 60-806 Poznan, Poland; 2 Department of Biotechnology and Food Microbiology, Poznan University of Life Sciences, 60-627 Poznan, Poland; 3 Department of Agronomy, Poznań University of Life Sciences, 60-632 Poznan, Poland

**Keywords:** active substances, antioxidants, diabetes mellitus, haskap berry leaves, inhibition of the enzyme, *Lonicera caerulea*

## Abstract

The presented research evaluates the medical use potential of *Lonicera caerulea* leaves, which are waste plants in cultivating berries. The study’s screening activity included the leaves of five varieties of *Lonicera caerulea*: Atut, Duet, Wojtek, Zojka, and Jugana. The microbiological analysis confirmed the safety of using *Lonicera caerulea* leaves without significant stabilization. *Lonicera caerulea* leaves standardization was carried out based on the results of the chromatographic analysis, and it showed differences in the contents of active compounds (loganic, chlorogenic and caffeic acids, and rutin), which are attributed to biological activity. For the *Lonicera caerulea* leaves varieties tested, the differences in the content of total polyphenol content, chlorophylls, and carotenoids were also confirmed. The screening of biological activity of five *Lonicera caerulea* leaf varieties was carried out concerning the possibility of inhibiting the activity of α-glucosidase, lipase, and hyaluronidase as well, and the antioxidant potential was determined. The defined profile of the biological activity of *Lonicera caerulea* leaves makes it possible to indicate this raw material as an essential material supporting the prevention and treatment of type II diabetes. However, this research showed that tested enzymes were strongly inhibited by the variety Jugana. The health-promoting potential of *Lonicera caerulea* leaves was correlated with the highest chlorogenic acid and rutin content in the variety Jugana.

## 1. Introduction

Haskap berry (*Lonicera caerulea* L.) belongs to the *Caprifoliaceae* family and is native to the Kamchatka area in Northeast Asia [1]. Blue honeysuckle fruits have been used in folk medicine [2]. Haskap berry has recently gained popularity thanks to its tasty and nutritionally rich fruits, now available in raw and dried form and preserves on world markets. Haskap berry fruits are characterized by a rich composition of active substances, including anthocyanins, phenolic acids, and a large amount of vitamin C [3,4,5,6]. The fruit of haskap berry, thanks to the composition of active substances, is characterized by a potent antioxidant and anti-inflammatory effect; moreover, the latest research indicates a high potential for their use in supporting the treatment of diabetes mellitus type 2 (DM2) [7,8,9,10,11]. *Lonicera caerulea* L. is a relatively little-researched plant material; however, all scientific research to date focuses on the activity of berries, which are used in the food industry; however, there is no particular attention paid to the rest parts of the plant.

Currently, we are observing a trend based on the reduction in generated waste and complete management of plant material due to the growing demand for food and the need to obtain the total value of the crops grown [12,13]. In the case of haskap berry crops, only fruits thoroughly tested in biological activity are used in the production. However, it should be remembered that active substances are present throughout the plant [14,15]. Haskap berry is a perennial plant that lives up to 35 years and can become a source of additional raw materials; however, obtaining them should not damage the bush itself [16]. For this reason, it seems reasonable to estimate the potential for the use of haskap berry leaves, the collection of which will not adversely affect the perennial itself.

The possibility of developing another part of the plant will allow greater use of the cultivated crops, generating potentially new pharmaceutical raw materials, which were treated as production waste. Due to the accumulation of different active substances in different parts of the plant, we do not necessarily have to observe overlapping or equally potent biological effects; however, studying unused raw materials seems crucial for developing novel natural-based compounds.

In addition, attention should be paid to the gaining popularity of the use of herbal infusions to support pharmacotherapy, particularly in the treatment of diabetes [17,18,19]. As a civilization disease that acts on many levels, it creates severe therapeutic problems [20,21]. Often, standard multi-level therapy is insufficient to control sugar levels, so patients benefit from raw materials of natural origin, often used as infusions [22]. In addition to controlling glucose levels in patients, a severe problem is often the co-occurring disease or side effects of constantly elevated blood sugar levels resulting from chronic inflammation in the body and the conditioning or exacerbation of symptoms caused by oxidative stress [23,24]. The conducted studies confirm that the administration of the extract in this way is a rich source of flavonoids and other polyphenols, which have a confirmed hypoglycemic effect, often showing at different levels of the glucose metabolic pathway, as well as showing high antioxidant activity [18,25]. Currently, the most common and most frequently used raw material in the form of infusions are white mulberry leaves; from the perspective of the wealth of plant raw materials, it seems necessary to look for new sources of raw materials that we will be able to administer in this form [26,27]. Therefore, the research aimed to determine the therapeutic potential of blue honeysuckle leaves as a new plant raw material with a specific action potential.

## 2. Materials and Methods

### 2.1. Plant Material

Leaf blades for analysis of 5 varieties (*Atut, Duet, Wojtek, Zojka, Jugana*) of *Lonicera caerulea* (haskap berry) were collected from shrubs grown on the plantation of the Research Centre for Cultivar Testing at the Experimental Station in Sulejów, the Department of Cultivar Testing in Masłowice (Łódź Voivodeship).

Five varieties of the haskap berry were cultivated on the soil of the excellent rye soil agricultural usefulness complex, quality class IVa, and soil abundance in available forms of nutrients was at an average level. The average air temperature was 8.7 °C, while the total atmospheric precipitation for the growing season was 542 mm. Leaf blades for analysis were collected during the planting period in the 3rd year of plantation management. The pH of the crop field was 5.8. The plantation was fertilized exclusively with manure at 25 tons per hectare. Shrubs in a row were grown 1.5 m apart; the distance between the rows was 3 m. Due to the lack of herbicide application on the plantation, the soil around the shrubs was covered with fine pine bark.

### 2.2. Materials

Standards of the determined substances: loganic acid, chlorogenic acid, caffeic acid and rutin were obtained by Sigma-Aldrich (USA).

Reagents used in conducted studies: α-D-glucopyranoside (PNPG), α-glucosidase, acarbose, 2,2-Diphenyl-1-picrylhydrazyl, TPTZ (2,4,6-tripyridyl-S-triazine) and Iron (III) chloride hexahydrate (FeCl_3_ × H_2_O), Folin–Ciocalteu’s phenol reagent, sodium carbonate, 2,4,6-tris(2-pyridyl)-1,3,5-triazine (TPTZ, C_18_H_12_N_6_), iron(III) chloride hexahydrate (FeCl_3_·6H_2_O), sodium chloride, bovine serum, hexadecyltrimethylammonium bromide (CTAB), hyaluronic acid (HA), Pancreatic lipase, Tris-HCL buffer, para-Nitrophenylphosphate (pNPP), Triton-X, sodium deoxycholate, gum Arabic were supplied by Sigma-Aldrich, St. Louis, MO, USA. Methanol, isopropanol, and acetone (Super Purity Solvent, Methanol 215 SPS) were supplied by ROMIL Ltd., Cambridge, England.

High-quality pure and ultra-high-quality pure water were prepared using a Direct-Q 3 UV Merck Millipore (Burlington, MA, USA) purification system.

Media used in microbiological assays: total count of aerobic bacteria (PCA, Merck, St. Louis, MO, USA), count of anaerobic bacteria (Schaedler Agar, Oxoid, Basingstoke, UK), count of yeasts and molds (YGC Agar, Oxoid), Enterobacteriaceae family bacteria count (VRBD, Merck, St. Louis, MO, USA), Escherichia coli count (TBX, Merck, St. Louis, MO, USA), Salmonella count (Chromogenic Salmonella Agar, Oxoid, Basingstoke, UK, temp. 37 °C).

### 2.3. Extract Preparation

Obtaining aqueous extracts from blue haskap berry leaves was preceded by grinding in a mill (2 times). Then, the raw material prepared this way was sieved through a 1 mm sieve to eliminate uncrushed leaf fragments. The extracts were prepared by weighing 1.0 g of the raw material into a flat bottom flask and adding 50 mL of distilled water obtained from the Direct-Q 3 UV Merck Millipore purification system (Merck, Darmstadt, Germany). The extraction was performed in a 30 °C ultrasonic bath (Bandelin Sonorex DT 1028 H; Bandelin, Germany, Berlin) for 60 min; the flask was kept off the light during the process. The ultrasonic bath was operated under predefined operating conditions at 35 kHz. The extracts were filtered through a filter paper to separate the remaining plant matrix from the extract. The extract prepared this way was divided into small portions and frozen at −20 °C to maintain the quantitative and qualitative composition throughout the research.

### 2.4. Microbiological Purity of the Haskap Berry Leaves

The microbiological quality of haskap berry leaves was determined using the standard plate count (SPC) method following the PN-ISO standards. Briefly, a portion of ground raw material (10.0 g) was weighed into sterile stomacher bags and homogenized with 90 mL sterile 0.1% *w*/*v* peptone buffered water (BPW) for 2 min. Serial 10-fold dilutions of each homogenate to 10^5^ were prepared in BPW. Portions of 0.1 mL from each dilution were spread on triplicate plates of PCA, Schaedler, YGC, VRBD, TBX, and Chromogenic *Salmonella* Agar. The total aerobic bacterial count (PCA), the count of anaerobic bacteria (Schaedler), the count of yeasts and molds (YPD), *Enterobacteriaceae* family bacteria count (VRBD), *Escherichia coli* count (TBX), and the count of bacteria belonging to the genus *Salmonella* (Chromogenic Salmonella Agar) were determined. Plates were incubated in conditions appropriate for a detected group of microorganisms: unlimited oxygen at 37 °C for 48 h (total aerobic count, *Enterobacteriaceae, Salmonella*), 44 °C for 48 h (Escherichia coli), 30 °C for five days (total yeast count) and in limited oxygen at 37 °C for 48 h (total anaerobic count). After incubation, typical colonies on the plates were enumerated, and the count of analyzed microorganisms in a 1.0 g sample was determined (CFU/g Dry leaves (DL))).

### 2.5. Determination of the Water Content

The analysis was performed using a moisture analyzer Ohaus MB120 (OHAUS Europe GmbH, Nänikon, Switzerland). 1.0 g sample was weighed into an aluminum tray and placed in the apparatus. Then, the sample was subjected to a drying process at 120 °C until a constant weight was obtained. The water content was expressed in % as the loss of the original sample weight in the heating process.

### 2.6. Determination of Total Phenolic Content (TPC)

TPC was determined using the Folin–Ciocalteu method with minor modifications. A 50 µL plant extract solution diluted 20 times was mixed with 50 µL of Folin–Ciocalteu reagent (F.-C.) and 100 µL distilled water. The mixture was pre-incubated for 5 min at 37 °C with shaking at 100 rpm. Then, 100 µL 20% Na_2_CO_3_ aq. the solution was added and incubated for 30 min at 37 °C with shaking at 100 rpm. The absorbance was read at 750 nm against the blank sample (water instead of the extract) in sixplicate. TPC was expressed as mg of gallic acid equivalent (GAE) per g of dry leaf mass utilizing a standard curve of gallic acid (y = 9.80847x − 0.2828; R^2^ = 0.9983) in the concentration range 0.06–0.2 mg/mL [28]. The content of TPC in the tested extract was calculated following the standard curve for gallic acid. The curve used to calculate the TPC content in the form of gallic acid as a conversion factor is presented in Supplementary Materials (Figure S1).

### 2.7. Chlorophyll and Carotenoids Content-Spectroscopic Determination

Chlorophyll A, B, and carotenoid content in haskap berries leaves was determined using the modified method described by Lichtenthaler et al. [29]. Briefly, 50.0 mg of homogenized dry leaves were incubated in 5.0 mL of 80% acetone and 20% 0,1% CaCO_3_ for 30 min in an ultrasonic bath at 30 °C. The obtained suspension was centrifuged to separate a portion of the solid leaf fragments. The obtained supernate was filtered through a 0.22 µm nylon syringe filter (Chemland, Stargard, Poland). Then, 100.0 µL of the resulting solution was added to a 96-well plate. Measurements were performed in *n* = 8 replicates, and the mean was calculated. The detections were performed at 470, 645, and 662 nm wavelengths using a Multiskan GO plate reader (Thermo Fisher Scientific, Vantaa, Finland).

The contents of chlorophyll A and B and carotenoids were calculated using the described equations [30,31,32,33]:

Chl a (mg/g DL):Ca = (11.24 Abs_662_ − 2.04 Abs_645_) × (*v*/*w*)

Chl b (mg/g DL):Cb = (20.13 Abs_645_ − 4.19 Abs_662_) × (*v*/*w*)

Total Carotenoids (mg/g DL) = [(1000 Abs_470_ − 1.90 Chl_a_ − 63.14 Chl_b_)/214] × (*v*/*w*)

where:

Absx—absorbance at x nm;
*v*—volume of solvent (mL);
*w*—the weight of the sample (mg);
DL—dry leaves.

### 2.8. Phenolic Acids Content-Chromatophical Determination

The HPLC analytical method was developed to determine phenolic compounds such as loganic acid, chlorogenic acid, caffeic acid, and rutin. The determinations were carried out using UHPLC Nexera (Shimadzu, Kioto, Japan) with the column: Zorbax SB-C18 (4.6 × 100 mm 3.5-Micron); the mobile phase contained 0.1% acetic acid (A) and acetonitrile (B). The developed method is based on the gradient elution following the scheme (%B): 0 min, 10%; 30 min, 20%; 60 min, 30%; 70 min, 45%; 70,1 min, 10%; followed by 10 min of washing the column with the starting phase. The phase flow was set at 1.0 mL/min, injection volume was 10.0 μL, and the column oven was set at 40 °C. Detections were carried out at multiple wavelengths: 210, 240, 254, 270, 320, and 380 nm.

### 2.9. Inhibition of In Vitro Activity of Enzymes

#### 2.9.1. Inhibition of α-Glucosidase

A modified spectrophotometric method developed by Telagari et al. [34] was used to determine the inhibition of α-glucosidase by the haskap berry leaves water extracts. Briefly, 50.0 μL of sample solution (118.8–277.2 µg mL^−1^ Atut; 206.0–370.8 µg mL^−1^ Deut; 208.0–370.4 µg mL^−1^ Wojtek; 204.0–367.2 µg mL^−1^ Zojka and 121.2–282.2 µg mL^−1^ Jugana) or acarbose (positive control, 1.0–5.0 mg mL^−1^) in different concentrations, the concentrations used are due to the different water content of the raw material and thus ultimately a different final maximum extract concentration, 50.0 μL of 0.1 M phosphate buffer (pH 6.8) and 30.0 µL α-glucosidase solution (1.0 U mL^−1^) was pre-incubated in 96 well plates at 37 °C for 15 min. Next, 20.0 μL of 5 mM p-nitrophenyl-α-D-glucopyranoside (pNPG) solution in a 0.1 M phosphate buffer (pH 6.8) was added incubated at 37 °C for 20 min. The reaction was terminated by adding 100.0 µL of sodium carbonate (0.2 M) into the mixture. The absorbance of the liberated p-nitrophenol was measured at 405 nm (Multiskan GO 1510, Thermo Fisher Scientific, Vantaa, Finland). The absorbance of enzyme solution without plant extracts/acarbose served as the control with total enzyme activity. The absorbance in the absence of the enzyme was used as the blind control. The enzyme inhibition rate expressed as a percentage of inhibition was calculated using the following formula:

$$\%\;inhibition\;activity=\left(\left(AC\;–AS\right)/AC\right)\times 100$$

where:

*AC*—the absorbance of the control (100% enzyme activity);
*AS*—the absorbance of the tested sample (haskap berries leaves water extract or acarbose).

Three independent experiments were carried out for the investigated substances, and the average from *n* = 8 measurements was calculated. Results were expressed as means ± S.D.

#### 2.9.2. Inhibition of Lipase

Inhibition of lipase by the aqueous extracts of haskap berries leaves was determined by a modified method described by Lewis and Liu [35]. Briefly, 20.0 µL of plant extracts (concentrations: 5.0; 10.0; 20.0 mg/mL) or positive control (Orlistat; 50.0 µg/mL) or negative control (water) was added to 96-well plates. Pancreatic lipase solution (10.0 mg/mL) was then freshly prepared in 50.0 mM Tris-HCL solution (pH 8 at 37 °C) and centrifuged to remove insoluble material. Then, 30.0 µL of enzyme solution and 100.0 µL of 50 mM Tris-HCl (pH 8) buffer were added to each well. After 20 min of incubation at 37 °C with continuous shaking at 200 RPM. A total of 150.0 µL of a substrate solution consisting of 30.0 mg pNPP dissolved in 10.0 mL isopropanol made up to 100.0 mL with 50.0 mM Tris-HCl buffer (pH = 8) containing 100.0 mg sodium deoxycholate, 50.0 mg gum arabic, and 1.0 mL Triton X was added and incubated with the same conditions for 30 min; The absorbance was then measured at 405 nm using Multiskan GO 1510, (Thermo Fisher Scientific, Vantaa, Finland). Three independent experiments were carried out for the investigated substances, and the average from *n* = 8 measurements was calculated. The percentage of inhibition was calculated by using the equation below.

$$\%\;inhibition\;activity=\left(1-\frac{ABS\;test-ABS\;blank}{ABS\;negative\;control-ABS\;blank}\right)\times 100\%$$

where:

*ABS test*—absorbance of sample;
*ABS blank*—absorbance of blank sample;
*ABS negative control*—absorbance of the negative control sample.

#### 2.9.3. Inhibition of Hyaluronidase

Inhibition of the aqueous extracts of haskap berry leaves was determined by the modified method described by hyaluronidase by Grabowska et al. [36]. Briefly, 25.0 µL of incubation buffer (50 mM, pH 7.0, with 77 mM NaCl and 1 mg/mL of bovine serum albumin), 25.0 µL of the enzyme (30 U/mL of acetate buffer pH 7.0), 10.0 µL solutions of the examined water extracts (5.0; 10.0 and 20.0 mg/mL), and 15.0 µL of acetate buffer (pH 4.5) were combined. The samples were incubated at 37 °C for 15 min. Next, 25.0 µL of HA (0.3 mg/mL in acetate buffer) was added. After incubation at 37 °C for 45 min, 200.0 µL of 0.325 g cetrimonium bromide (CTAB) dissolved in 18.0 mL of 2% NaOH was added to undigested HA precipitated. The turbidity of the reaction mixture was measured as the absorbance at 600 nm (Multiskan GO 1510, Thermo Fisher Scientific, Vantaa, Finland) after 10 min of incubation at room temperature. Kaempferol was used as the positive control (0.2–1.0 mg/mL). Three independent experiments were carried out for the investigated substances, and the average from *n* = 8 measurements was calculated. The percentage of Inhibition was calculated by using the equation below:

$$\%\;inhibition\;activity=\left(Ts-TEblank\right)\left(THblank-TEblank\right)\times 100\%$$

where:

*TS*—absorbance of sample;
*TEblank*—absorbance of the enzyme + examined substance;
*THblank*—absorbance of the HA + examined substance.

### 2.10. Antioxidant Action

#### 2.10.1. DPPH Assay

The DPPH assay was effected according to Studzińska-Sroka et al. with modifications [37]. Briefly, 25.0 μL of the aqueous extracts of haskap berry leaves was dissolved in distilled water at different concentrations (79.2–158.4 mg mL^−1^ Atut; 80.8–161.6 mg mL^−1^ Deut; 83.2–166.4 mg mL^−1^ Wojtek; 81.6–163.2 mg mL^−1^ Zojka; and 82.4–164.8 mg mL^−1^ Jugana) and was mixed with 175.0 μL of DPPH (Sigma-Aldrich, St. Louis, MO, USA) solution (3.9 mg in 50 mL of MeOH). The reaction mixture was shaken and incubated in the dark at room temperature for 30 min. The control contains 25.0 μL of distilled water and 175.0 μL of DPPH solution. Absorbance was measured at 517 nm. The inhibition of the DPPH radical by the sample was calculated according to the following formula:

DPPH scavenging activity (%) = (A0 − A1)/A0 ×100%

where:

A0—the absorbance of the control
A1—the absorbance of the sample.

#### 2.10.2. FRAP Assay

According to Tiveron et al. [38], the FRAP assay was performed with some modifications. The stock solutions of FRAP reagent included 300 mM acetate buffer (pH 3.6), 10 mM TPTZ solution in 40 mM HCl, and 20 mM FeCl_3_·6H_2_O solution. The working FRAP solution was prepared by mixing 25.0 mL of acetate buffer, 2.5 mL of TPTZ solution, and 2.5 mL of FeCl_3_·6H_2_O solution and then warmed at 37 °C before usage. Briefly, 25.0 μL of the tested extracts dissolved in distilled water at different concentrations (0.2–1.0 mg/mL) were mixed with 175.0 μL of FRAP solution, shaken, and incubated at 37 °C for 30 min. in the dark condition. Then, the absorbance was read at 593 nm. The results were expressed as the IC_0.5_, corresponding to the extract concentration required to produce a 0.5 O.D. value.

## 3. Results and Discussion

Four Polish varieties of haskap berries (Atut, Duet, Wojtek, and Zojka), as well as one variety of Jugana of Russian origin, were tested. All varieties used in the research are characterized by the possibility of industrial use due to the excellent plant habit and abundant production of fruits, which are the main product used in the food industry.

Despite the high value attributed to the Kamchatka berry fruit, which is characterized by high anti-inflammatory activity and antioxidant potential, the fruit has also found application in potential diabetes therapy thanks to the ability to inhibit enzymes responsible for the breakdown of carbohydrates into simple sugars [39,40,41]. Leaves of haskap berry, a new potential source of biologically active compounds, have not been tested so far. The leaves obtained after harvesting were dried at 40 °C for two days to reduce the potential decomposition of active substances in the tested plant material [42].

An essential aspect of the value of using plant raw materials is their microbiological quality. It is essential for raw materials that can be taken in simple technological preparations (e.g., dried leaves in the form of tea to be infused) [43,44]. Moreover, the microbiological status of plant raw material is essential because treatments to remove microorganisms from it are usually costly and may result in a reduction in the content of active substances in it [45]. Therefore, our first research aimed to determine the microbiological quality of the raw leaves of the haskap berry. The obtained results were compared with the requirements presented by the European Pharmacopoeia (EP), the United States Pharmacopoeia (USP), and the requirements specified by the World Health Organization (WHO), which are presented in Table S1 [43]. Contamination of the tested samples with aerobic bacteria ranged between 2.0 × 10^2^ and 6.4 × 10^3^ CFU/g. Concurrently, the total count of aerobic bacteria in the tested material was similar to that of anaerobic bacteria. Contamination of the tested samples with yeasts and molds was low at the level of 2.0 × 10^1^–3.2 × 10^2^ CFU/g. *E. coli, Salmonella*, and other *Enterobacteriaceae* were not detected in any samples. Therefore, the obtained results showed that the tested plant raw material was of high microbiological quality and suitable for further technological processing. According to the EP and WHO, this raw material can be used without the addition of boiling water. However, the requirements of the USP suggest the need to treat it with hot water before direct use due to exceeding the acceptable standards for aerobic bacteria (>10^2^ CFU/g) and mold and yeast (>10 CFU/g). It is worth emphasizing that the tested plant material was not treated to any initial technological process, such as washing, but only dried at 40 °C. However, due to the variability of the tested material’s composition and the possibility of environmental factors’ effect on its quality, it is recommended to control the microbiological purity of each batch of raw material or consider implementing simple methods to increase the tested material’s microbiological quality [46,47]. The results of the microbiological quality assessment are presented in Table 1 as CFU/g DL.

Because of the high microbiological quality of the tested material, the absence of mold, and the lack of antimicrobial (toxic) activity, it was decided not to test the content of aflatoxins. However, it should be remembered that this is one of the critical criteria for approving a product batch on the market [48,49,50]. Due to the severe health consequences of potential consumption, the WHO estimates that about 25% of the world’s production of plant food products must be destroyed annually due to aflatoxin contamination [51].

To initially evaluate the quality of the examined haskap berry leaves, the raw material was tested for the sum of polyphenolic compounds in the obtained aqueous extracts and the content of chlorophyll A and B and carotenoids in the leaf itself. Moreover, the water content in the dried leaves was examined using a moisture analyzer. The water content in the samples tested was in the range of 5.43–6.38% after drying the raw material. Preliminary content analysis showed significant differences between the haskap berry varieties tested. The highest content of TPC was demonstrated for the variety Jugana (52.399 mg/g DL), while the lowest content was obtained for the variety Wojtek (28.179 mg/g). A high correlation between TPC and then the antioxidant activity of the raw material has been demonstrated many times; therefore, after the initial screening tests of the content, we can conclude the high antioxidant potential of the tested raw material and the fact that the Jugana variety as with the highest TPC content will be characterized by the highest ability to scavenge free radicals [52,53].

Moreover, differences in the content of chlorophyll and carotenoids were observed; however, they were less significant, but once more, the best results were obtained for the Jugana variety and the lowest for the Wojtek variety (Table 2). So far, no haskap berries for chlorophyll content analysis have been carried out, but a similar analysis has been carried out for *Lonicera japonica*, belonging to the same genus [54]. The results indicate a significantly higher level of chlorophyll in the leaves of Kamchatka berry because in the case of chlorophyll A it is about three-fold and about two-fold for chlorophyll B. However, it should be noted that the results are only illustrative due to the high variability between species, the significant influence of the environment, and, more specifically, sunlight, affecting the chlorophyll content. For the content of carotenoids, the analysis in the leaves of the haskap berry was not carried out. However, an analogous analysis was carried out for the flowers of two cultivars in different development cycles, indicating about 10 to 5 times higher leaf content [55]. However, as in the case of chlorophyll content, the number of carotenoids can be significantly affected by environmental conditions; however, genome analysis indicates the ability of the *Lonicera* family to synthesize them [56,57].

The tests were repeated after six months. Samples were stored at 25 °C and 60% humidity with no light exposure. Re-testing aimed to determine the drop in the quality of the tested raw material during storage and the ability to absorb water to determine the potential storage conditions. The obtained results are presented in Table 2.

The assumption was made of a change of at least 10% from the initial value as the limit value allowing the raw material to be considered stable during storage. Under the tested conditions, the obtained results meet the adopted criteria. However, we observe a loss of tested substances and an increase in water content in the tested samples, which allows us to believe that it is necessary to use packaging limiting the access of moisture to the product to limit degradation over time and to accumulate water and thus to potential development pathogenic microorganisms [58,59,60].

Composition analysis was performed using the HPLC-DAD method to obtain a broader spectrum of active substances in the tested plant material. The study standardized the raw material (Table 3) for selected active substances (loganic acid, chlorogenic acid, caffeic acid, and rutin) present in all varieties with confirmed biological activity [61,62]. The selection of the compounds determined in detail was based on their high biological activity and the fact that they were excellent analytical markers of the changes taking place due to the size of the observed peak in the analysis. The highest content for the selected standards was demonstrated for the Jugana variety, while the lowest for the Wojtek variety and Zojka for the loganic acid content. The obtained results correlate with the initial evaluation of the raw material, presenting the Jugana variety as having the highest content of active substances, thus potentially having the highest biological activity [63,64].

The study was also repeated in the tested conditions after six months. The criterion for recognizing stability was also used by maintaining at least 90% of the content of the active compound compared to the initial sample. This criterion was met for all variants as well as for all tested active compounds. Thus, the high stability of selected active compounds of the raw material during the storage period was confirmed.

The plant-derived acids (loganic, chlorogenic, and caffeic acids) selected in the analysis are characterized by high biological activity, particularly concerning the ability to inhibit selected enzymes, e.g., a-glucosidase, which is essential in the development and course of type 2 diabetes due to its ability to reduce absorbed simple sugars. In particular, chlorogenic acid is demonstrated by high activity against the indicated enzymes, which is present in large amounts in the tested plant material (0.987 mg/g DL for the Jugana variety) [65]. Loganic acid is also connected with anti-inflammatory properties and can reduce hyperlipidemia [66]. Caffeic acid is responsible for the anti-inflammatory and antioxidant activity; despite its low content in the raw material, due to the coverage of biological effects, it will potentially have an entourage effect enhancing the activity of the whole extract; in addition, it can inhibit enzymes responsible for digesting simple sugars. However, it shows a higher response to α-amylase than to α-glucosidase [67]. On the other hand, rutin has a broad spectrum of biological activity, but mainly the ability to scavenge free radicals, which will translate into a high antioxidant potential of the raw material [68].

To estimate the biological activity potential of water extracts obtained from haskap berry leaves, the inhibitory activity was determined with selected enzymes in vitro models. As part of the research, an α-glucosidase inhibition assay was used to determine the antidiabetic potential [69,70], a pancreatic lipase inhibition assay was used to determine the hypolipidemic potential [71,72], and a hyaluronidase inhibition assay was used to determine the anti-inflammatory potential of the raw material [73,74]. Moreover, the antioxidant potential of the extracts was determined in two independent models (DPPH and FRAP) to complete the biological activity profile of the new raw material. The obtained results are presented in Table 4 [75,76]. The presented results refer to the base concentration of the extract used without considering the reagents used in a given model.

**Figure 1:**
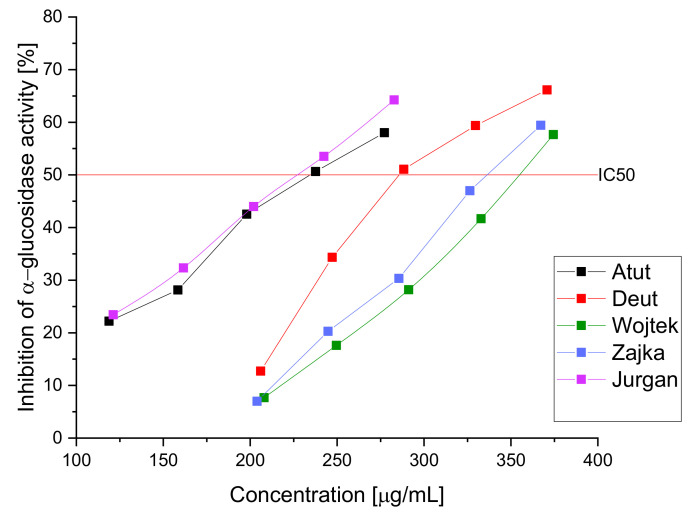
Inhibition of α-glucosidase activity by aqueous extracts of haskap berry leaves.

As a result of the α-glucosidase inhibition study, a high inhibitory potential of the extracts (IC_50_ ranging from 227.43 ± 2.98 to 354.45 ± 4.01 µg/mL) was demonstrated (Figure 1); the obtained results indicate a more substantial effect of the aqueous extract than the acarbose used in the reference test (IC 50: 3573.22 ± 5.47 µg/mL). Previous studies indicated the high hypoglycemic potential of the haskap berry fruit through potent inhibition of α-glucosidase and α-amylase; however, the leaves have not yet been studied as the potential raw material with potent inhibitions against α-glucosidase [11]. The high antidiabetic activity of the raw material cannot be strictly attributed to the content of a given active compound due to the presence of the so-called entourage effect responsible for enhancing the effect of the extract through all its components. However, attention should be paid to the high content of chlorogenic acid, which is characterized by the ability to inhibit a-glucosidase. Demonstrating activity in the remaining unused plant fragments seems attractive due to the possibility of using them in dietary supplements or a tea additive with a hypoglycemic effect. Natural raw materials are becoming increasingly popular to fight against the first symptoms of civilization diseases and support traditional pharmacotherapy. Patients will receive most plant materials due to their low cost and good biological response; however, it should be remembered that they will not fully replace pharmacotherapy and can only be used as a fulfillment.

**Figure 2:**
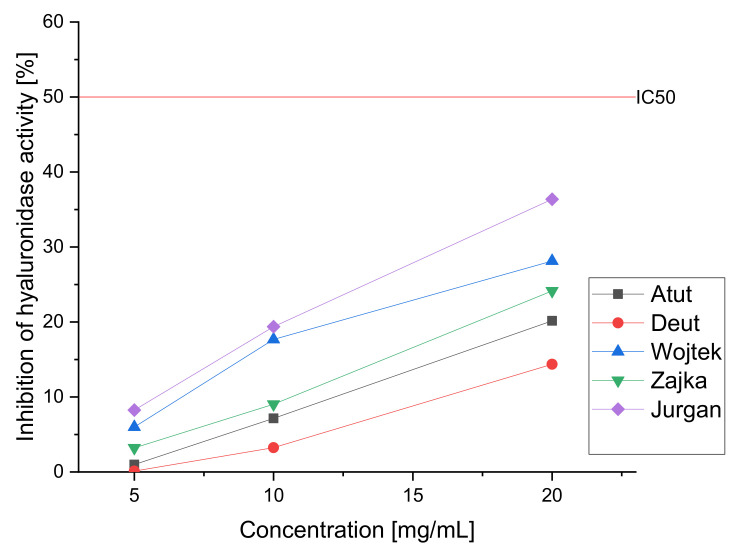
Inhibition of lipase activity by aqueous extracts of haskap berry leaves.

To estimate the potential hypolipidemic effect due to the ability to inhibit pancreatic lipase by the obtained aqueous extracts of haskap berry leaves, a study was carried out in which the original extract and its subsequent diluents (20.0, 10.0, and 5.0 mg/mL) were used to estimate the lipase inhibition capacity of the water extract and thus determine the hypolipidemic potential (Figure 2). In the study, the inhibition of the enzyme at the level of at least 50% (IC_50_) was not obtained. However, the inhibition of the enzyme is observed to a lesser extent; moreover, we can observe significant differences between the tested cultivars. The highest activity is observed again in the Jugana variety (32,87% of enzyme inhibition for 20.0 mg/mL extract); the literature indicates the lipase inhibitory effect by loganic acid, the content determined using the HPLC, was the highest in this cultivar. The raw material can be classified as showing a low hypolipidemic effect. However, it should be noted that the model used is based solely on the digestive enzyme secreted into the intestine. So far, studies have been performed only based on extracts obtained from fruit, which showed anti-obesity and liver protective activity and inhibited fat accumulation in the liver [77,78,79,80]. The obtained results allow us to conclude about the possible hypolipidemic effect of the raw material; however, to estimate the full potential, the effect of the raw material on intracellular enzymes and signaling pathways should be investigated.

**Figure 3:**
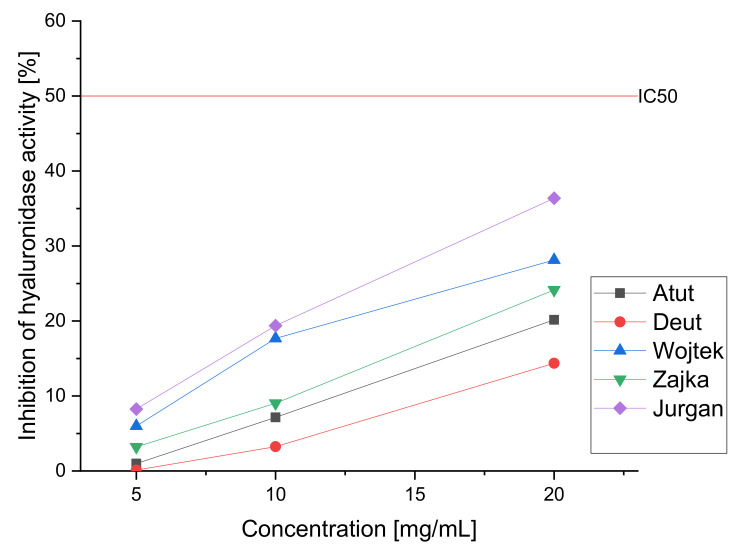
Inhibition of hyaluronidase activity by aqueous extracts of haskap berry leaves.

To assess anti-inflammatory activity study was carried out to determine the ability to inhibit hyaluronidase by aqueous extracts of haskap berry. The prepared aqueous extracts (20.0, 10.0, and 5.0 mg/mL) were tested in vitro (Figure 3). The obtained results indicate the ability to inhibit the enzyme; however, 50% inhibition was not observed even at high concentrations, allowing the determination of the IC_50_ value. As in the case of other enzyme studies, we observe significant differences between the cultivars. As in the previous tests, the Jugana variety showed the highest activity (36,37% of enzyme inhibition for 20.0 mg/mL extract) due to the highest content of active compounds. However, one should remember the simplicity of the applied in vitro test, which is natural screening. The research using compounds obtained from the haskap berries has proven their effectiveness in reducing pro-inflammatory cytokines [39].

**Figure 4:**
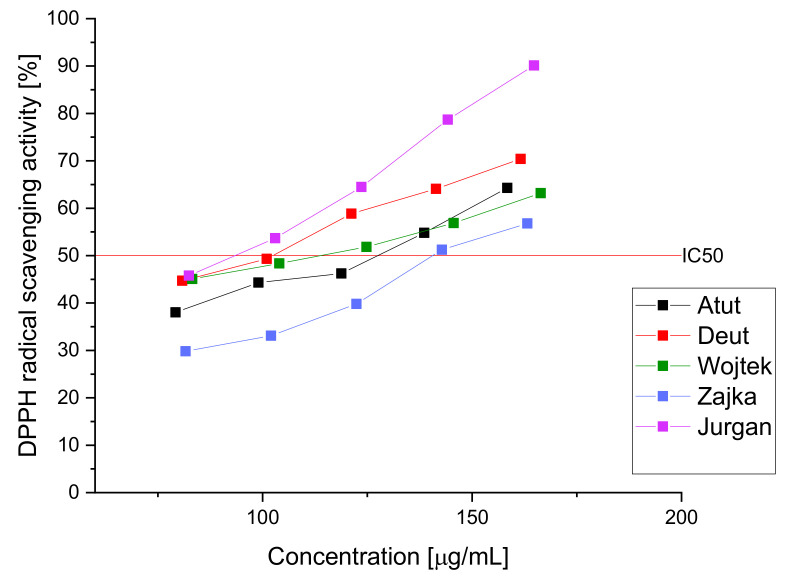
DPPH radical scavenging activity of aqueous extracts of haskap berry leaves.

**Figure 5:**
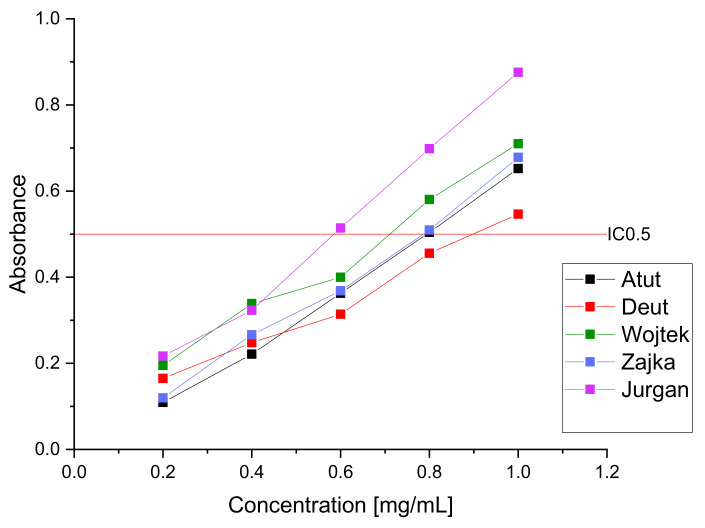
Antioxidative activity of aqueous water extracts of haskap berry leaves in FRAP model.

To estimate the activity of the tested raw material, the obtained extracts were tested in the DPPH and FRAP assays to determine the raw material’s antioxidant potential (Figure 4 and Figure 5). The obtained results indicate high antioxidant activity, which can be attributed to the significant content of polyphenolic compounds such as chlorogenic acid or rutin [68,81]. The obtained results indicate a similar antioxidant activity of the haskap berry cultivars examined leaves. However, we observe the advantage of the Jugana (DPPH IC_50_-93.36 μg/mL and FRAP IC_0.5_-0.59 mg/mL, respectively) cultivar resulting from the higher content of the sum of active compounds influencing the antioxidant potential, especially in higher concentrations of the extracts used.

An essential aspect of the activity of plant materials is their ability to scavenge and neutralize free radicals, which generate an inflammatory reaction by generating oxidative stress.

The marked high antioxidant activity seems significant due to the tested plant material’s demonstration of the biological activity profile to lower blood sugar levels. In type 2 diabetes, we observe an increasing production of free radicals that condition damage to the beta islets of the pancreas and cause increased insulin resistance of tissues [23,82,83]. The combination of activity inhibiting the absorption of simple sugars and providing protection by scavenging free radicals classifies the raw material as desirable for use in supporting treatment and preventing the origin of DM2 [84].

## 4. Conclusions

All the tested varieties of *Lonicera caerulea* meet the microbiological criteria of WHO, FDA, and EU for raw materials used without pre-treatment with boiling water while maintaining USP requirements. The storage tests were carried out for six months, indicating no need to apply special storage conditions.

The screening of enzyme inhibition of α-glucosidase, lipase, and hyaluronidase indicated the most active variety-Jugana, which was also characterized by the highest content of active substances (loganic acid, chlorogenic acid, caffeic acid, and rutin as well as total polyphenols content). However, it should be noted that all the tested cultivars were characterized by significant biological and antioxidant activity in the in vitro models used.

The studies indicate the possibility of using *Lonicera caerulea* leaves as infusions to support the therapy of type 2 diabetes. The raw material has a pleiotropic effect, starting from inhibiting enzymes responsible for the absorption of simple sugars and helping to lower cholesterol, which is often a problem in overweight patients. The simultaneous anti-inflammatory and antioxidant effect will potentially reduce the complications of diabetes and, at the same time, alleviate the further course of the disease due to the inhibition of pro-inflammatory processes, stimulating the cascade development of further complications.

The obtained results confirm the high potential for medicinal use of *Lonicera caerulea* leaves in the form of simple infusions as a new plant raw material. In order to assess the full spectrum of biological activity and the optimal route of administration, further studies will be needed to explore the mechanisms of activity further. In addition, potential pre-formulation work will allow for the optimal delivery of active compounds contained in the raw material.

## Figures and Tables

**Table 1:** Microbiological purity of haskap berry leaves.

Samples	Counts of Microorganisms (CFU/g DL **)					
	AB *	ANB *	MY *	En *	*E.coli*	Sal *
Atut	5.1 × 10^3^	5.5 × 10^3^	2.1 × 10^2^	n/f	n/f	n/f
Duet	9.2 × 10^2^	9.9 × 10^2^	3.0 × 10^1^	n/f	n/f	n/f
Wojtek	3.5 × 10^2^	3.8 × 10^2^	2.0 × 10^1^	n/f	n/f	n/f
Zojka	2.0 × 10^2^	2.9 × 10^2^	5.0 × 10^2^	n/f	n/f	n/f
Jugana	6.4 × 10^3^	5.2 × 10^3^	3.2 × 10^2^	n/f	n/f	n/f

* AB–aerobic bacteria; ANB–anaerobic bacteria; MY–mold and yeast; EN–*Enterobacteriaceae* and other Gram-negative bacteria: *E.coli*–*Escherichia coli*; Sal–*Salmonella*; n/f–not found; ** DL–dry mass of leaves material.

**Table 2:** Quantitative evaluation of haskap berry leaves at the begging and after 6 months.

Variety	TPC (mg/g DL *)		Chlorophyll A (mg/g DL *)		Chlorophyll B (mg/g DL *)		Carotenoids (mg/g DL *)		Water Content (%)	
	T = 0 Months	T = 6 Months	T = 0 Months	T = 6 Months	T = 0 Months	T = 6 Months	T = 0 Months	T = 6 Months	T = 0 Months	T = 6 Months
Atut	49.618 ± 1.352	47.494 ± 1.471	11.659 ± 0.511	11.401 ± 0.131	4.540 ± 0.121	4.398 ± 0.141	1.956 ± 0.084	1.901 ± 0.061	5.43 ± 0.32	5.66 ± 0.15
Duet	44.893 ± 2.344	43.146 ± 0.782	12.753 ± 0.449	12.483 ± 0.097	5.484 ± 0.096	5.331 ± 0.135	2.163 ± 0.095	2.096 ± 0.023	5.75 ± 0.41	5.97 ± 0.18
Wojtek	28.179 ± 0.983	27.072 ± 0.761	10.642 ± 0.387	10.293 ± 0.190	4.463 ± 0.174	4.309 ± 0.136	1.848 ± 0.117	1.807 ± 0.021	6.23 ± 0.37	6.48 ± 0.33
Zojka	32.127 ± 1.085	31.223 ± 0.752	13.382 ± 0.533	12.902 ± 0.122	5.632 ± 0.153	5.474 ± 0.152	2.217 ± 0.126	2.150 ± 0.041	6.56 ± 0.49	6.84 ± 0.40
Jugana	52.399 ± 1.730	50.956 ± 0.971	14.003 ± 0.312	13.673 ± 0.097	5.575 ± 0.113	5.409 ± 0.243	2.876 ± 0.081	2.806 ± 0.097	6.38 ± 0.37	6.59 ± 0.22

* DL–Dry mass of leaves material; Data expressed as mean ± SD.

**Table 3:** Results of the content of active substances by HPLC for haskap berry leaves in T = 0 and T = 6 months.

Variety	Loganic Acid (mg/g DL *)		Chlorogenic Acid (mg/g DL *)		Caffeic Acid (mg/g DL *)		Rutin (mg/g DL *)	
	T = 0 Months	T = 6 Months	T = 0 Months	T = 6 Months	T = 0 Months	T = 6 Months	T = 0 Months	T = 6 Months
Atut	2.511 ± 0.121 *	2.498 ± 0.103 *	0.879 ± 0.112 *	0.804 ± 0.107 *	0.039 ± 0.003 *	0.035 ± 0.002 *	0.730 ± 0.243 *	0.708 ± 0.222 *
Duet	1.870 ± 0.151 *	1.801 ± 0.125 *	0.618 ± 0.157 *	0.578 ± 0.121 *	0.019 ± 0.002 *	0.017 ± 0.001 *	0.519 ± 0.353 *	0.498 ± 0.334 *
Wojtek	1.764 ± 0.183 *	1.705 ± 0.091 *	0.345 ± 0.104 *	0.309 ± 0.070 *	0.045 ± 0.003 *	0.041 ± 0.003 *	0.125 ± 0.274 *	0.111 ± 0.207 *
Zojka	0.803 ± 0.156 *	0.801 ± 0.103 *	0.389 ± 0.156 *	0.366 ± 0.123 *	0.056 ± 0.001 *	0.055 ± 0.002 *	0.143 ± 0.196 *	0.140 ± 0.204
Jugana	2.981 ± 0.172 *	2905 ± 0.150 *	0.987 ± 0.073 *	0.924 ± 0.097 *	0.078 ± 0.002 *	0.070 ± 0.003 *	0.879 ± 0.124 *	0.877 ± 0.113

* Dry mass of leaves material; Data expressed as mean ± SD.

**Table 4:** In vitro activity of haskap berry leaves water extracts.

Cultivar	IC_50_ (µg/mL)	For an Extract with a Concentration of 20 mg/mL	
	Inhibition of α-Glucosidase	% of Inhibition of Lipase	% of Inhibition of Hyaluronidase
Atut	234.53 ± 3.35 *	21.26 ± 1.32 *	20.16 ± 1.77 *
Duet	285.76 ± 2.84 *	15.21 ± 1.11 *	14.37 ± 1.65 *
Wojtek	354.45 ± 4.01 *	26.26 ± 2.01 *	28.14 ± 1.43 *
Zojka	336.23 ± 3.07 *	22.78 ± 1.87 *	24.14 ± 2.03 *
Jugana	227.43 ± 2.98 *	32.87 ± 1.56 *	36.37 ± 1.67 *
	**IC_50_ (µg/mL)**	**IC_0.5_ (mg/mL)**	
	**DPPH**	**FRAP**	
Atut	121.04 ± 2.11 *	0.79 ± 0.08 *	
Duet	102.42 ± 4.33 *	0.89 ± 0.04 *	
Wojtek	113.77 ± 1.79 *	0.71 ± 0.05 *	
Zojka	140.57 ± 1.24 *	0.77 ± 0.07 *	
Jugana	93.36 ± 2.18 *	0.59 ± 0.02 *	

Data expressed as mean ± SD; * significance with *p* ≤ 0.05.

## Data Availability

The data are contained within the article or Supplementary Materials.

## Supplementary Materials

The following supporting information can be downloaded at: https://www.mdpi.com/article/10.3390/nu14193898/s1, Table S1. Microbiological purity standards for plant raw materials, Figure S1. The calibration curve for gallic acid used to determine the TPC content.
