# Supplementary material for: Haskap Berry Leaves (Lonicera caerulea L.)—The Favorable Potential of Medical Use
Source: Nutrients. 2022 Sep 21;14(19):3898. doi: 10.3390/nu14193898 (PMC9573050; doi:10.3390/nu14193898)
Supplement: Supplementary file 1 [file nutrients-14-03898-s001.zip › nutrients-1916464-supplementary.pdf]

nutrients

MDPI

Article

Haskap Berry Leaves (*Lonicera caerulea* L.)—The Favorable Potential of Medical Use

Szymon Sip [1], Anna Sip [2], Piotr Szulc [3] and Judyta Cielecka-Piontek [1,]*

1 Department of Pharmacognosy, Faculty of Pharmacy, Poznań University of Medical Sciences, 60-806 Poznań
2 Department of Biotechnology and Food Microbiology, Poznan University of Life Sciences, 60-627 Poznań, Poland
3 Department of Agronomy, Poznań University of Life Sciences, 60-632 Poznań, Poland
* Correspondence: jpiontek@ump.edu.pl

Citation: Sip, S.; Sip, A.; Szulc, P.; Cielecka-Piontek, J. Haskap Berry Leaves (*Lonicera caerulea L.*)—The Favorable Potential of Medical Use. *Nutrients* **2022**, *14*, 3898. https://doi.org/10.3390/nu14193898

Academic Editor: Adam Matkowski

Received: 30 August 2022
Accepted: 15 September 2022
Published: 21 September 2022

Publisher's Note: MDPI stays neutral with regard to jurisdictional claims in published maps and institutional affiliations.

Copyright: © 2022 by the authors. Licensee MDPI, Basel, Switzerland. This article is an open access article distributed under the terms and conditions of the Creative Commons Attribution (CC BY) license (https://creativecommons.org/licenses/by/4.0/).

Table S1. Microbiological purity standards for plant raw materials.

	European Pharmacopoeia[1]	United States Pharmacopoeia[2]	WHO[3]
**Aerobic bacteria**	10^7 / 10^5	10^5 / 10^4 / 10^2	* / 10^7 / 10^5
**Mold and yeast**	10^5 / 10^4	10^3 / 10^2 / 10	10^5 / 10^4 / 10^3
**Enterobacteriaceae**	* / 10^3	103 / * / *	* / 10^4 / 10^3
**E. coli**	10^3 / absent	Absent	10^4 / 10^2 / 10
**Salmonella**	* / absent	Absent	* / absent /absent

* Limits are not specified. 1 - European Pharmacopoeia: Herbal medicinal products consisting exclusively of one or more herbal medicines (whole, reduced, or powdered): the first value indicates herbal medicinal products to which boiling water is added before use; The second value is herbal medicinal products to which boiling water is not added before use. 2 - United States Pharmacopoeia: The first value represents dried or powdered botanicals and botanicals that must be soaked in boiling water before use; The second value is tinctures, powdered plant extracts, liquid extracts, and plant dietary supplements; The third value represents infusions/decoctions. 3 - WHO: The first value is the limit of contamination for "raw" plant material intended for further processing; The second value is for plant materials that have been pretreated (e.g., boiling water); the third value is for other plants material for internal use.

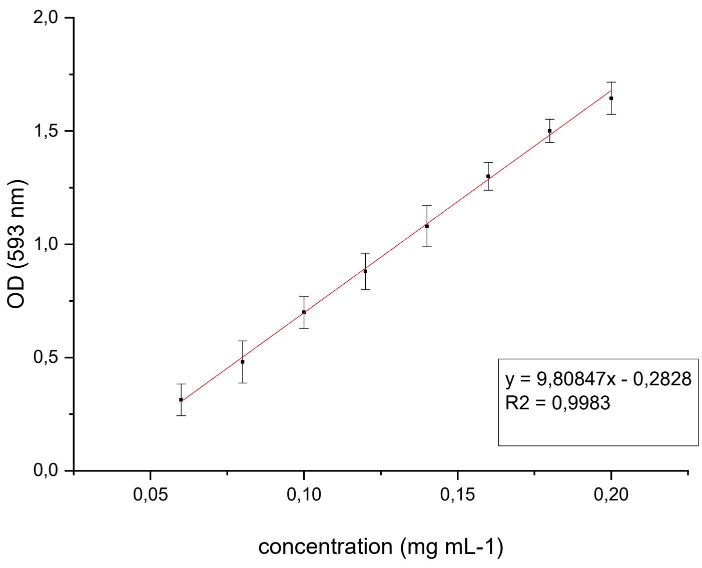

Figure S1. The calibration curve for gallic acid used to determine the TPC content.
